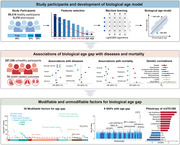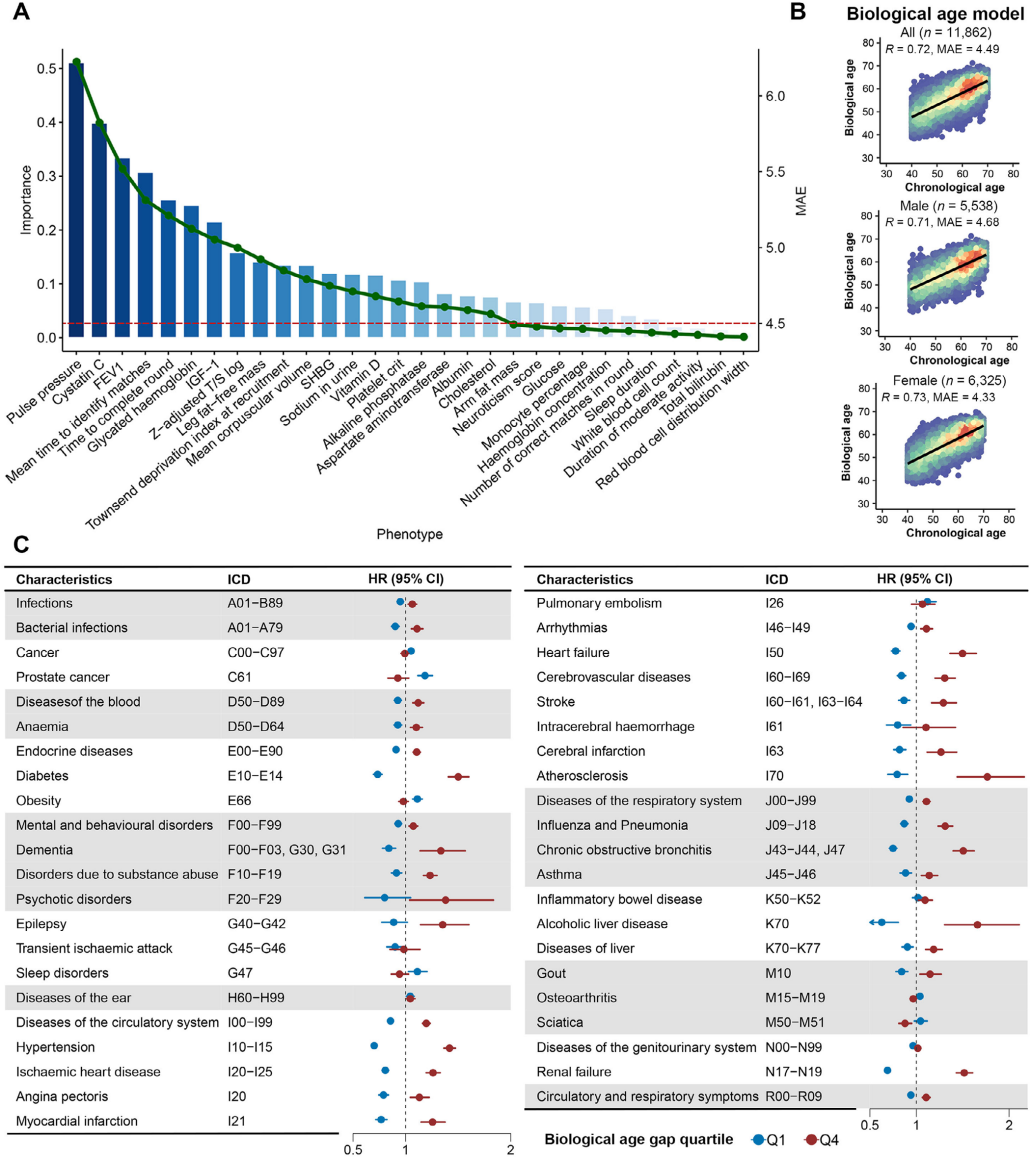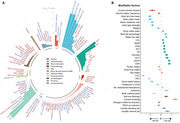# Association of biological age with health outcomes and its modifiable factors

**DOI:** 10.1002/alz.088206

**Published:** 2025-01-09

**Authors:** Jin‐Tai Yu

**Affiliations:** ^1^ Huashan hospital, Fudan University, Shandong, China; National Center for Neurological Disorders, Shanghai, Shanghai, China; Huashan Hospital, Fudan University, Shanghai, Shanghai, China; National Center for Neurological Disorders, Shanghai China

## Abstract

**Background:**

Identifying the clinical implications and modifiable and unmodifiable factors of aging requires the measurement of biological age (BA) and age gap.

**Method:**

Leveraging the biomedical traits involved with physical measures, biochemical assays, genomic data, and cognitive functions from the healthy participants in the UK Biobank, we establish an integrative BA model consisting of multi‐dimensional indicators.

**Result:**

Accelerated aging (age gap >3.2 years) at baseline is associated incident circulatory diseases, related chronic disorders, all‐cause, and cause‐specific mortality. We identify 35 modifiable factors for age gap (p < 4.81 × 10^−4^), where pulmonary functions, body mass, hand grip strength, basal metabolic rate, estimated glomerular filtration rate, and C‐reactive protein show the most significant associations. Genetic analyses replicate the possible associations between age gap and health‐related outcomes and further identify CST3 as an essential gene for biological aging, which is highly expressed in the brain and is associated with immune and metabolic traits.

**Conclusion:**

Our study profiles the landscape of biological aging and provides insights into the preventive strategies and therapeutic targets for aging.